# Surgical approach for appendicitis in neutropenia: a case report and review of the literature

**DOI:** 10.1186/s40792-022-01563-x

**Published:** 2022-11-17

**Authors:** Ushanthan Uthayanan, Walter Kolb, Bruno Schmied, Stephan Bischofberger

**Affiliations:** Department of General, Visceral, Endocrine and Transplant Surgery, Kantonsspital St. Gallen, Saint Gall, Switzerland

**Keywords:** Appendicitis, Neutropenia, G-CSF, Appendectomy, Case report

## Abstract

**Background:**

Acute appendicitis is a common abdominal pathology, particularly in younger patients presenting with abdominal pain. The clinical presentation is typically characterized by right lower quadrant pain (with local peritonitis) accompanied by fever and nausea. In neutropenic patients it is challenging to diagnose acute appendicitis. It is much more challenging because the characteristic symptoms are different, and diagnosis may be delayed or missed.

**Case presentation:**

We present the case of a 33-year-old Caucasian male patient with fever, abdominal pain, and an absolute granulocyte count of 0 × 10^9^/L. Abdominal CT demonstrated an uncomplicated acute appendicitis. We initiated a conservative in-hospital treatment with intravenous antibiotic therapy and simultaneous bone marrow stimulation, with close monitoring. On day three, there was evidence of monocyte increase, one of the first signs of bone marrow regeneration, and delayed laparoscopic appendectomy was performed. The perioperative and postoperative course was uneventful.

**Conclusion:**

We discuss the different treatment strategies in patients with neutropenia presenting with acute appendicitis (i.e., conservative management, delayed appendectomy, and immediate appendectomy) based on our experience and a review of the literature. In summary, delayed laparoscopic appendectomy at the onset of granulocyte regeneration under antibiotic and G-CSF therapy represents a viable surgical option for adults as well as for children and should be discussed compared with conservative therapy.

## Background

Neutropenia is defined as an neutrophil count of less than 1.5 × 10^9^/L. It is caused by several factors: chemotherapy, drug reactions, severe sepsis and immunodeficiency syndromes. In approximately 30% of neutropenic patients with fever caused by infections, the origin is located in the gastrointestinal tract. The main cause is neutropenic enterocolitis, whereas acute appendicitis is a more minor cause, affecting 5% of cases only [1].

Abdominal surgery in neutropenic patients is challenging, particularly in an emergency setting. To make the decision between surgical and non-surgical management is difficult, because the patients history, his clinical presentation, and the diagnostic findings differ from the general population.

For patients with neutropenia with suspected acute appendicitis, there are no clear treatment recommendations or guidelines available [2, 3]. This is a problem because the number of immunosuppressed patients has increased recently due to modern medicine with numerous chemotherapeutic agents. Additionally, secondary myelosuppression because of systemic infections or primary diseases of the hematopoietic system are possible etiologies [1].

This case report presents an adult patient with acute appendicitis and neutropenia most likely caused by secondary myelosuppression and the safe approach of delayed appendectomy under granulocyte colony-stimulating factor (G-CSF) stimulation.

## Case presentation

We present the case of a 33-year-old Caucasian male patient. His main complaints were fever, chills, extremity pain, sore throat, and right lower quadrant abdominal pain. Relevant secondary diagnoses included Hodgkin’s disease, which was treated with the BEACOPP chemotherapy regimen (bleomycin, etoposide, doxorubicin, cyclophosphamide, vincristine, procarbazine, and prednisone) in 2015 and is currently in remission. Additionally, the patient has a known human immunodeficiency virus (HIV) infection classified as stage A2 in the Centers for Disease Control and Prevention classification system [4], which is currently treated with antiviral therapy; the patient is currently without viral detection.

**Fig. 1:**
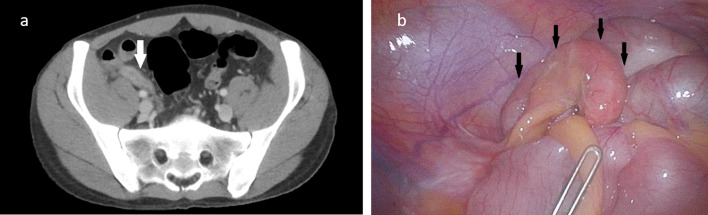
Radiological and laparoscopic features of acute appendicitis in a patient with neutropenia. Abdominal computed tomography showed appendicitis with phlegmonous inflammatory reaction surrounding the appendix without abscess or appendicolith (**a**, white arrow). Laparoscopically, the appendix vermiformis appears phlegmonously altered, but not purulent (**b**, black arrows)

In the clinical examination, the patient presented with tachycardia, mild hypotension, fever (39 °C), and tenderness on palpation in the right lower abdomen without peritoneal irritation. Other significant physical findings were enlarged pharyngeal tonsils with stippling and slightly enlarged right-sided cervical lymph nodes. Laboratory findings showed an anemia with a hemoglobin count of 117 g/L [140–180 g/L], a neutropenia with an absolute granulocyte count of 0.0 × 10^9^/L [1.4–8.0 × 10^9^/L], leukocyte count of 1.0 × 10^9^/L [4–10 × 10^9^/L], and a C-reactive protein of 281 mg/L [< 8 mg/L]. Computed tomography (CT) of the neck/thorax/abdomen revealed an appendicitis with phlegmonous inflammatory reaction surrounding the appendix without abscess or appendicolith (Fig. 1a). The pathologically enlarged cervical lymph nodes were probably due to tonsillitis. Otherwise, there was no CT evidence of the recurrence of Hodgkin’s disease. Further serial laboratory tests for Epstein–Barr virus infection, coronavirus disease infection, and HIV load are negative. Neutropenia is therefore not due to an active HIV infection, Hodgkin’s disease or other infection. Normal leukocyte counts in the past also make chronic bone marrow toxicity after chemotherapy seven years ago unlikely. In summary, the diagnosis was an uncomplicated appendicitis acuta with neutropenia and fever. Neutropenia was likely associated with acute abdominal inflammation due to secondary myelosuppression.

**Fig. 2:**
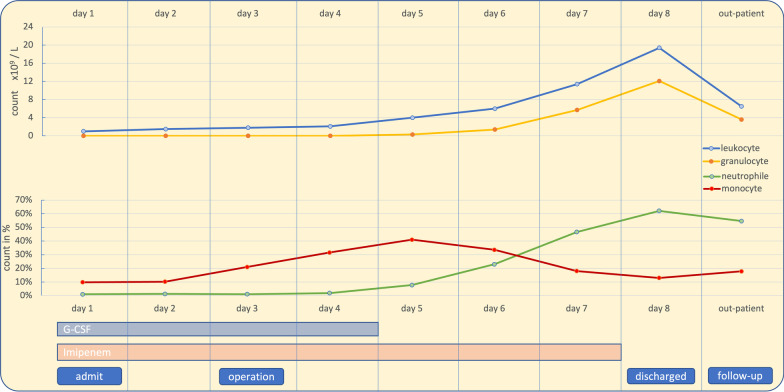
Graphical presentation of white blood cell values and duration of G-CSF and imipenem therapy. On day three, there was evidence of monocyte increase, indicator representing the onset of granulocyte regeneration. The delayed laparoscopic appendectomy was performed at day three. G-CSF therapy was discontinued on the first postoperative day, and imipenem was stopped on the fourth postoperative day, with already normalized granulocyte count. Patient was discharged on the fifth postoperative day. In the out-patient follow-up, leukocyte count remained normal

We have established a multidisciplinary treatment strategy (general medicine, oncology, and surgery) (Fig. 2). We started a conservative in-hospital treatment with intravenous antibiotic therapy (500 mg imipenem four times daily) and simultaneous bone marrow stimulation (30 million units of G-CSF once a day). The patient was monitored closely. On day three, there was evidence of monocyte increase (from 9 to 21% [up to 41%] [reference range, 2–12%]) under G-CSF, an indicator representing the onset of granulocyte regeneration, while the absolute granulocyte count remained at 0.0 × 10^9^/L and CRP remained stagnant at 262 mg/L. A Follow-up CT showed no improvement or worsening of the findings. Clinically, abdominal pressure pain and fever remained without rebound tenderness.

Because of the onset of bone marrow regeneration and the lack of clinical and imaging improvement with broad-spectrum empirical antibiotic therapy, laparoscopic appendectomy was performed. No perioperative complications occurred. Intraoperatively, a phlegmonously altered non-purulent appendix was observed (Fig. 1b). Histopathological examination showed a moderately chronic, mild acute appendicitis. The clinical symptoms returned rapidly to normal with persisting mildly enlarged tonsillitis without signs of inflammation; thus, the G-CSF therapy was discontinued on the first postoperative day, and imipenem was stopped on the fourth postoperative day. We discharged the patient in good general condition on the fifth postoperative day. In the out-patient follow-up, leukocyte count remained normal (6.5 × 10^9^/L), and a regular wound healing was observed.

## Discussion

In an immunocompetent patient, appendicitis presents with right lower abdominal pain and rebound tenderness. The symptoms are caused by peritoneal irritation because of the inflammatory changes in the appendix vermiformis with fibrin and pus production. Especially, neutrophil granulocytes suffer cell death when “fighting” against the infection [5]. In neutropenic patients, these granulocytes are missing; therefore, the induction of fibrin and pus formation is insufficient. Consequently, the classic clinical signs may be absent because of the lack of peritonism, which delays the diagnosis and treatment of appendicitis [6]. Diagnosis can be confirmed using sonography or abdominal CT. Neutrophilic enterocolitis is one of the most important differential diagnoses.

Appendicitis in infants or younger children with neutropenia is rare. Two retrospective studies have discussed this issue. In a retrospective multicenter study from France, published in 2017, 30 children with appendicitis and neutropenia were analyzed [7]. Among the 30 children, 20% underwent primary surgery, and 56% underwent interval surgery after the initiation of antibiotic therapy. No life-threatening complications occurred in both groups; however, wound infection was observed in 30% of the children in the primary surgery group. Only 23% of the children could be treated conservatively. Similar results were obtained in a retrospective multicenter study from the USA involving 66 children undergoing cancer treatment, published in Pediatrics 2021 [8]. Of the 66 children, 41% underwent surgery for appendicitis in neutropenia, and 56% were treated conservatively with antibiotics and ± G-CSF. However, 46% of the conservatively treated children required surgery in the same hospitalization period due to the failure of conservative therapy (65%) or recovery of granulocytes (35%), and another 13% in the interval. The complication rate was significantly reduced in the operative group compared with that in the non-operative group. Children in the operative group had a shorter hospitalization duration and less delay in the chemotherapy regimen.

The afore-mentioned studies showed that delayed surgical therapy with appendectomy combined with antibiotic therapy and ± G-CSF therapy is a viable option in children. Similar considerations were made by Sullivan et al. in their review published in Oncology considering the results in children [9]. Source control in a patient with neutropenia is paramount, which can be achieved either by appendectomy or by drainage in the case of relevant abscesses.

Studies in adult patients are rare like the observational study involving 32 adult patients with cancer, appendicitis, and neutropenia. The authors reported that 25% of the patients received appendectomy without relevant complications, 62.5% were treated conservatively, and 12.5% were treated by a drain [10]. The interval appendectomy rate in the conservative group was high at 30% (50% as elective and 50% as emergency). The failure rate in the drainage group was also remarkable at 25%. Despite the high failure rate, the authors recommend conservative therapy rather than surgery [1, 10].

In uncomplicated appendicitis, conservative therapy with intravenous antibiotic therapy alone can be discussed in selected immunocompetent patients according to the study by Moris et al. [11]. However, the study also points out the risk of treatment failure, especially in cases of appendiceal dilatation (≥ 7 mm) or presence of appendicolith. Patients with an appendicolith should be mandatorily referred to surgery [2] because of a high failure rate of up to 60% reported in the conservative therapy arm [12]. Even without the presence of these risk factors the recurrence rate in immunocompetent patients is already 39% [2] and will consequently be significantly higher in immunosuppressed patients.

The prior/simultaneous application of G-CSF in this therapy regimen is controversial and has been described in various case reports [13, 14]. The success of bone marrow stimulation with G-CSF is initially shown by an increase in monocytes, which undergo a faster differentiation from the myeloid stem cells than neutrophil granulocytes [15]. Additionally, there is probably delayed granulocyte recovery in the laboratory because the newly formed granulocytes are directly consumed by the active inflammation. There is a risk that the consumption of granulocytes will lead to fulminant inflammation with consecutive perforation of the appendix, which is the main complication of conservative therapy. In contrast, neutrophil granulocytes are essential for adequate wound healing, which should be considered and may prefer a delayed surgical treatment therapy. The surgical approach can significantly reduce the rate of wound infection. The incidence of wound infection was significantly higher in open appendectomy (7.6%) than in laparoscopic appendectomy (0%) [16].

**Fig. 3:**
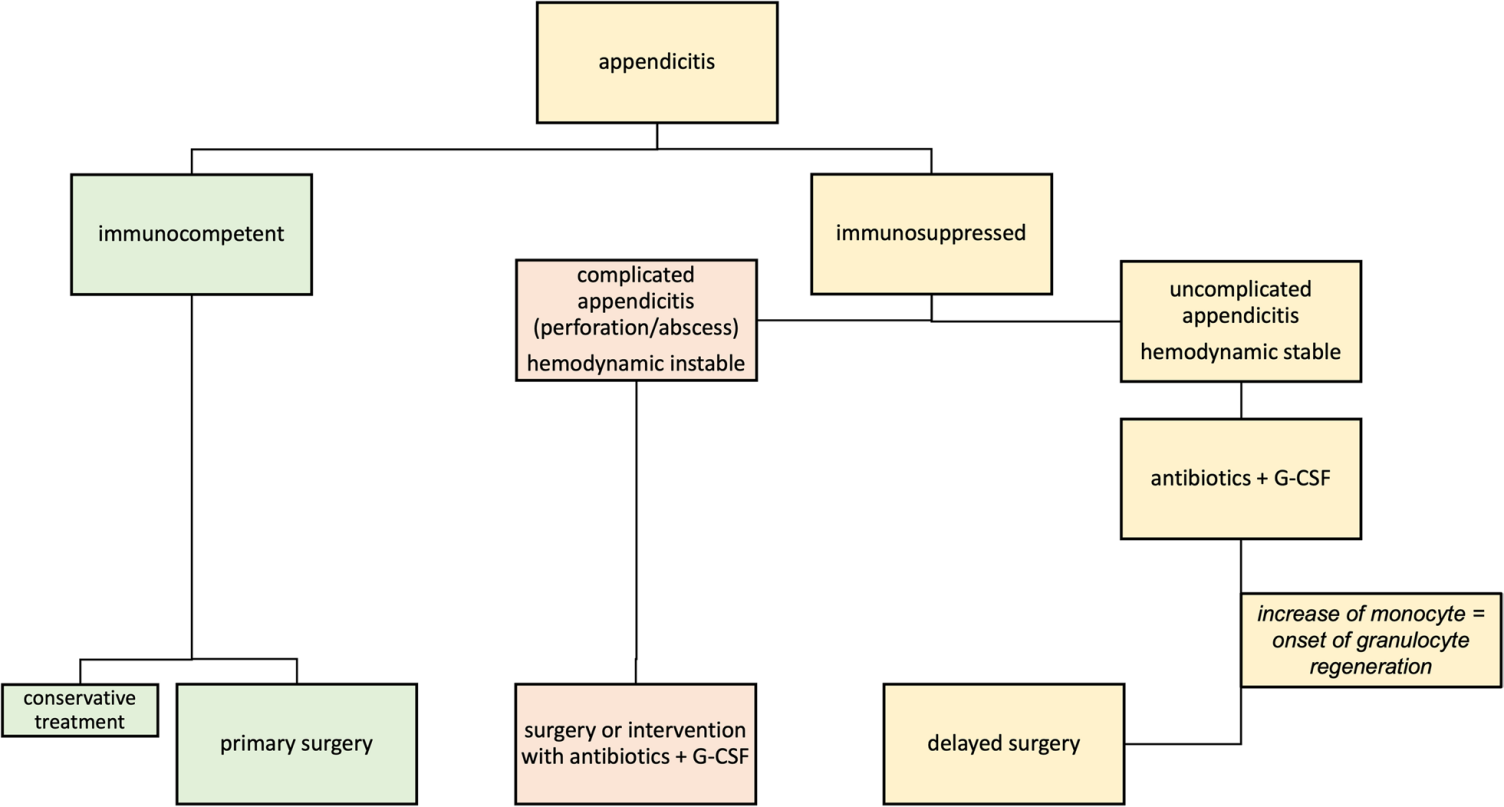
Therapy algorithm—appendectomy is the treatment of choice for acute appendicitis in immunocompetent patients. A conservative therapy with intravenous antibiotic therapy alone can be discussed in selected patients. Immunosuppressed patients divided in uncomplicated or complicated appendicitis. Septic patients with/without complicated appendicitis should always be evaluated for immediate surgical or interventional therapy, accompanied by antibiotic therapy and other supportive therapies. For a clinically stable patient presenting with an uncomplicated appendicitis, we recommend initially antibiotic and G-CSF therapy. At the onset of granulocyte regeneration, shown in the evidence of monocyte increase, the delayed appendectomy should be performed, for an adequate wound healing

In this case report, we initially started with antibiotic and G-CSF therapy in a hemodynamically stable patient. We characteristically detected an increase in the monocyte count on the third hospitalization day and decided to perform laparoscopic appendectomy on the same day. The aim was to prevent the fulminant inflammation on the one hand and to use the upcoming regeneration of granulocytes for wound healing on the other hand (see flowchart Fig. 3). Histopathological examination of the appendix of the presented patient revealed acute-on-chronic inflammation, so that conservative therapy would probably have failed in our patient.

We recommend surgical therapy in immunosuppressed patients, based on this case report and review of literature, because in all studies discussed here [7–10], the failure rate of conservative therapy was high and surgery was finally required. The conservative therapy with antibiotics followed by surgical therapy due to therapy failure prolongs the patient's convalescence time. A pending chemotherapy in tumor patient must therefore be paused longer, which may affect the prognosis of the patient.

The described therapy regimen is only a feasible option for a clinically stable patient presenting with an uncomplicated appendicitis (without perforation and abscess) and is the limitation of this case report. Septic patients should always be evaluated for immediate surgical or interventional therapy, accompanied by antibiotic therapy and other supportive therapies.

## Conclusion

Laparoscopic appendectomy is the treatment of choice for acute appendicitis in immunocompetent patients. The surgical therapy in immunosuppressed patients revealed the wound infections as relevant surgical complications, so that in our opinion a delayed laparoscopic appendectomy under antibiotic and G-CSF therapy should be performed in the same hospital stay instead of an immediate surgery. The optimal time for surgery is at the onset of granulocyte regeneration, shown in the evidence of monocyte increase, for an adequate wound healing. In case of missing the optimal time the risk of provoking a complication such as abscess or perforation are increased due to the recovery of granulocytes, which are the main causes for failure of conservative therapy.

Surgery represents a viable option for adults as well as for children and should be discussed compared with conservative therapy with a higher failure rate, various complications, and the necessity of interval appendectomy and an prolonged convalescence time.

## Data Availability

All data generated or analysed during this study are included in this published article.

## Abbreviations

CRP: C-reactive protein
CT: Computed tomography
G-CSF: Granulocyte colony-stimulating factor
HIV: Human immunodeficiency virus
Fig.: Figure

## References

[1] White MG, Morgan RB, Drazer MW, Eng OS (2021). Gastrointestinal surgical emergencies in the neutropenic immunocompromised patient. J Gastrointest Surg.

[2] Di Saverio S, Podda M, de Simone B, Ceresoli M, Augustin G, Gori A (2020). Diagnosis and treatment of acute appendicitis: 2020 update of the WSES Jerusalem guidelines. World J Emerg Surg.

[3] Gorter RR, Eker HH, Gorter-Stam MAW, Abis GSA, Acharya A, Ankersmit M (2016). Diagnosis and management of acute appendicitis. EAES consensus development conference 2015. Surg Endosc..

[4] Reimer J, Franke GH, Ross B, Gerken G, Heemann U. HIV-Infektion: CDC-Klassifikation und Lebensqualität. In: HIV-Infekt: Springer, Berlin, Heidelberg; 2000. p. 751–756. doi:10.1007/978-3-642-59683-4_133.

[5] Pisetsky DS (2011). Pus: the Rodney Dangerfield of immunology. Arthritis Res Ther.

[6] Scott-Conner CE, Fabrega AJ (1996). Gastrointestinal problems in the immunocompromised host. A review for surgeons. Surg Endosc.

[7] Scarpa AA, Hery G, Petit A, Brethon B, Jimenez I, Gandemer V (2017). Appendicitis in a neutropenic patient: a multicentric retrospective study. J Pediatr Hematol Oncol.

[8] Many BT, Lautz TB, Dobrozsi S, Wilkinson KH, Rossoff J, Le-Nguyen A (2021). Appendectomy versus observation for appendicitis in neutropenic children with cancer. Pediatrics.

[9] Sullivan PS, Moreno C (2015). A multidisciplinary approach to perianal and intra-abdominal infections in the neutropenic cancer patient. Oncology (Williston Park).

[10] Santos D, Chiang Y-J, Badgwell B (2016). Appendicitis in cancer patients is often observed and can represent appendiceal malignancy. Am Surg.

[11] Moris D, Paulson EK, Pappas TN (2021). Diagnosis and management of acute appendicitis in adults: a review. JAMA.

[12] Mahida JB, Lodwick DL, Nacion KM, Sulkowski JP, Leonhart KL, Cooper JN (2016). High failure rate of nonoperative management of acute appendicitis with an appendicolith in children. J Pediatr Surg.

[13] Kang HJ. Treatment of acute appendicitis in the patients with severe neutropenia. J Med Cases. 2013. 10.4021/jmc1286w.

[14] Ustun C (2007). Laparoscopic appendectomy in a patient with acute myelogenous leukemia with neutropenia. J Laparoendosc Adv Surg Tech A.

[15] Sugimoto Y, Katayama N, Masuya M, Miyata E, Ueno M, Ohishi K (2006). Differential cell division history between neutrophils and macrophages in their development from granulocyte-macrophage progenitors. Br J Haematol.

[16] Marzouk M, Khater M, Elsadek M, Abdelmoghny A (2003). Laparoscopic versus open appendectomy: a prospective comparative study of 227 patients. Surg Endosc.

